# Do Not Hallow until You Are out of the Wood! Ultrasonographic Detection of CPP Crystal Deposits in Menisci: Facts and Pitfalls

**DOI:** 10.1155/2013/181826

**Published:** 2013-07-16

**Authors:** Georgios Filippou, Antonella Adinolfi, Panagiotis Bozios, Sauro Lorenzini, Valentina Picerno, Valentina Di Sabatino, Ilaria Bertoldi, Dario Gambera, Mauro Galeazzi, Bruno Frediani

**Affiliations:** ^1^Department of Medicine, Surgery and Neurosciences, Rheumatology Section, University of Siena, Policlinico le Scotte, Viale Bracci, 53100 Siena, Italy; ^2^Orthopedic Unit, Casa di Cura Rugani, SS 222 Chiantigiana, 53100 Siena, Italy

## Abstract

*Purpose*. Ultrasonography (US) has been demonstrated to be an important tool in the diagnosis of calcium pyrophosphate (CPP) crystal deposition disease. The aim of our study was to individuate and describe possible pitfalls in US detection of such deposits in menisci. *Patients and Methods*. We enrolled all patients waiting to undergo knee replacement surgery due to osteoarthritis, for one-month period. Each patient underwent US examination of the knee, focusing on the menisci. After surgery, the menisci were examined by US, macroscopically and microscopically, using the microscopic analysis as the gold standard for CPP deposition. *Results*. 11 menisci of 6 patients have been studied. Ex vivo examination of menisci performed better in CPP identification than in vivo examination. The possible reasons of misinterpretation or misdiagnosis of the in vivo exam were identified and are extensively described in the paper. Also a new sign of CPP crystal deposits was found. *Conclusions*. This study permitted to highlight some difficulties in CPP crystal detection by US in menisci. Further studies are needed to define completely US CPP crystal aspect and to improve the sensibility and specificity of US in CPP deposition diagnosis.

## 1. Introduction

**Figure 1:**
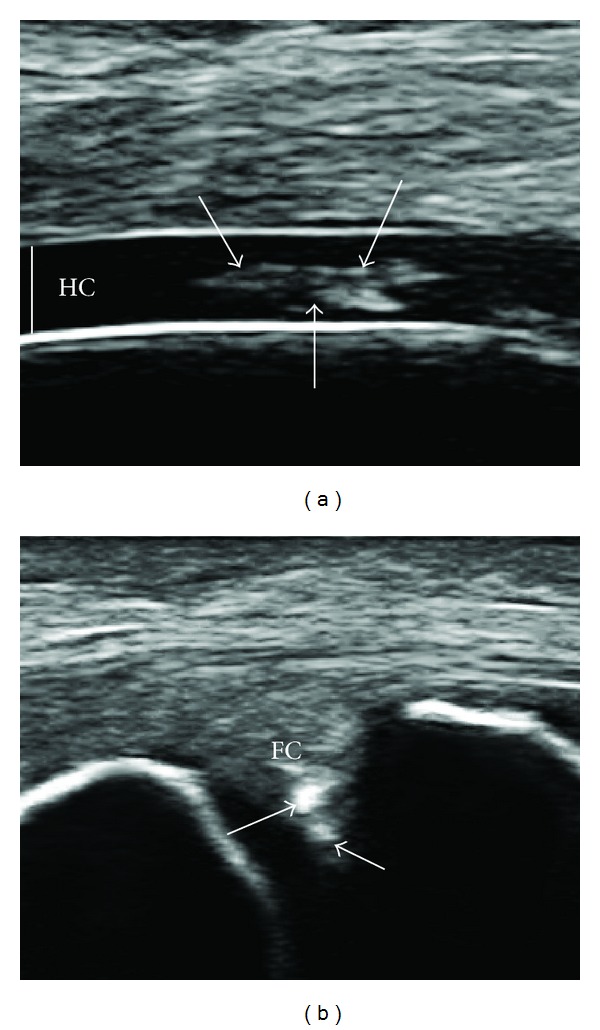
US aspect of CPPD deposits (arrows) in the hyaline cartilage (HC) of the femur in a knee joint and in the fibrocartilage (FC) of a medial meniscus.

Over the last decade, ultrasonography (US) has been demonstrated to be an excellent technique for detecting calcium pyrophosphate dihydrate (CPP) crystal deposits in joints and periarticular tissues [1–8], and the aspect of these deposits in hyaline cartilage and fibrocartilage has been described (Figure 1). In the literature the sensitivity of US in identifying CPP crystal deposits varies between a minimum of 15% [3] for the plantar fascia and a maximum of 89% [7] for the hyaline cartilage of the knee. In contrast, the specificity of US remains at constantly high values, in excess of 90%. In these studies, the gold standard for CPP disease diagnosis was the McCarty criteria [9] or microscopic synovial fluid analysis. According to the McCarty criteria, a patient should only receive a definite diagnosis if typical crystal deposits are seen at plain radiography and synovial fluid analysis. If only one of these two criteria is satisfied, then the diagnosis is probable but not definite. This makes it difficult to create a clear classification of patients who are affected by the pathology or not, as there is some evidence that plain radiography may not reveal calcium pyrophosphate dihydrate deposition (CPPD) visible by US [4, 10]. Furthermore crystal deposits in the joint are not stable, and it has been demonstrated that CPP deposits decrease at X-ray following a pseudogout attack, according to the crystal shedding theory [9]. However, in a recent study of our group we demonstrated that the adoption of a rigorous gold standard for the detection of CPP crystals in menisci can lead to the reduction of sensibility and specificity values of US [11]. This could be due to the presence of alterations in fibrocartilage tissue that could create false positive or negative findings on US examination. 

The objective of our study was to further define the US aspect of CPP crystal deposits in human menisci, in vivo and ex vivo, using polarized light microscopy of menisci samples as the gold standard and to describe the possible pitfalls that could lead to false positives or negatives during in vivo examination.

## 2. Patients and Methods

We enrolled in our study all consecutive patients waiting to undergo knee replacement surgery due to severe osteoarthritis at the orthopedic clinic of the University of Siena, for one-month period. Before enrollment, patients gave informed consent for the study. All patients were required to undergo US examination of the knee on the day before surgery. Only the knee subjected to surgery was examined by an expert ultrasonographer. US scans were only performed at the level of the medial and lateral meniscus, with the knee completely extended, semiflexed, and completely flexed, without raising the probe all the way along the medial and lateral rim. No other joint structures were examined, and the sonographer did not ask the patients any questions, in order to prevent clinical data influencing the judgment of US findings. The sonographer gave a dichotomous score based on the absence/presence of CPP deposits in the meniscus, according to the previously published criteria [4]. 

The day after US examination, the patients underwent total knee replacement surgery performed by an expert orthopedic surgeon. The patients' menisci were extracted and placed in a jar with saline solution in a refrigerator at 4°C. A day or two after surgery, the menisci were collected and examined. At this stage, a third rheumatologist examined the menisci macroscopically and photographed each meniscus from both sides. The same sonographer then reexamined the menisci at the end of the study period, without knowing the name of the patient. All the menisci were immerged in bath of gel and examined with longitudinal and transverse scans. The sonographer again gave a dichotomous score based on the absence/presence of CPP deposits, according to the same criteria used for the in vivo analysis. When the sample was positive, he indicated the exact position in which a sample should be collected for microscopic analysis, by making a US-guided cut on the surface of the meniscus. An Esaote Mylab 70XVG (Esaote, Florence, Italy) scanner equipped with 7–18 MHZ linear probe has been used for this study. 

In the third phase, all the menisci were examined by a biologist and expert in synovial fluid analysis, who prepared slides. A small sample was collected from the surface of every meniscus, along with a sample from the point indicated by the sonographer. In the case of negative ex vivo US, four random samples were collected from each meniscus: one from the anterior horn, one from the mid-portion, one from the posterior horn, and one from the surface. All slides were then examined by a rheumatologist who is an expert in synovial fluid analysis and was blinded to the US findings. Each slide was observed under transmitted light microscopy with the condenser diaphragm placed as close as possible to enhance the refractivity of the crystals (“pseudophase” lighting), and by compensated polarized microscopy. At least 40 adjacent microscopic fields were carefully scanned on each slide. Previously published criteria have been used for CPP crystal identification and differential diagnosis with basic calcium crystals and steroid deposits [9, 12, 13]. Each slide was judged as positive or negative for CPPD crystals. Microscopic analysis of the specimens was considered as the gold standard for the diagnosis.

## 3. Results 

We enrolled 6 patients in our study, 5 females and 1 male. The mean age was 78 years (range 63–92 years). In one case the lateral meniscus could not be entirely retrieved during surgery so it was excluded from the study. We finally examined 11 menisci: 6 medial and 5 lateral.

In a previous study, we explained the results on US sensibility and specificity for in vivo and ex vivo examinations. US demonstrated a sensitivity of 44% and specificity of 50% in the in vivo study, compared to 67% and 100%, respectively, in the ex vivo study. In the samples collected after the ultrasound-guided cut, CPP crystals were found in all cases [11].

**Figure 2:**
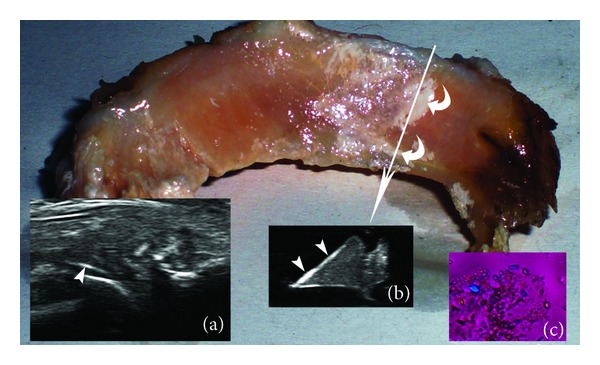
Superficial CPP deposits in the meniscus (curved arrows). Frame (a): the appearance of these deposits in the in vivo examination (arrowheads). Frame (b): ex vivo US transverse scan of the meniscus at the level of the arrow. The arrowheads highlight a hyperechogenic line on the surface of the meniscus, corresponding to the CPP deposits. Frame (c): microscopic analysis of a sample of the meniscus surface, confirming the presence of CPP crystals.

The previously described punctuated pattern of CPP deposits has been found in menisci fibrocartilage and corresponds to CPP deposits as confirmed by microscopic analysis. In some cases, fibrous tissue could resemble this pattern and give a false positive result; this aspect is analyzed later in the Discussion. Another interesting finding is that CPP deposits are present not only in the context of fibrocartilage, but also on the surface of the menisci. In microscopic analysis, in 9 out of 11 menisci, CPP crystals have been found in the specimen of the meniscus surface while internal CPP deposits have been found in 8 meniscus. In ex vivo US examination hyperechoic deposits on the meniscus surface, suggestive of the presence of CPP deposits have been found in 5 menisci. In these cases an hyperechogenic enhancement of the meniscus surface has been found (Figure 2), due to the presence of the CPP deposits. In four cases, US did not show any appreciable alteration of meniscus surface probably because CPP crystals were too few to be visualized on US. In in vivo examination such sign was present (Figure 2) but has been observed and considered only retrospectively, reevaluating previously acquired images, as this is the first time that this sign is being described. Recently we observed the same hyperechogenic line on the cartilage surface of a patient affected by CPPD [14], resembling the “double contour” sign, as described for patients affected by gout [7].

## 4. Discussion

### 4.1. General Considerations on US Sensitivity and Specificity in CPPD Disease

Over the last decade US has been used increasingly for the diagnosis of CPPD in the joints, and the main US findings have been described extensively by us and other authors [1–8]. The main US landmark of the disease is the presence of hyperechoic deposits, not creating posterior shadowing, in the hyaline cartilage and in the fibrocartilage of joints (menisci, triangular fibrocartilage of the wrist) (Figure 1). All the studies that have sought to assess US in the detection of CPPD disease have been performed on patients with definite disease [1–3, 7, 8], according to the McCarty criteria [9], or have used the presence of CPP crystals in synovial fluid as the gold standard [4–6]. Almost 6 years ago, Sanchis and Pascual demonstrated that CPP crystals are also commonly found in asymptomatic patients [15]. However, Schlesinger et al. [16] recently found that 13.5% of the synovial fluids initially found to be positive for the presence of CPP crystals were negative at subsequent analysis. In the first study all patients enrolled were affected by CPPD and no control group was used, while the second was a retrospective analysis. On the other hand, plain X-rays have shown a sensitivity of 39% for detecting chondrocalcinosis [17], and it has been demonstrated that MRI is insensitive to the presence of CPPD in the knee, even when these deposits are widespread [18].

Recently, a group of experts has published on behalf of the EULAR a set of recommendations for the calcium pyrophosphate deposition disease regarding terminology, diagnosis, and management of the disease [10]. The experts, after systematic review of the literature and based mainly on our previous papers on the use of US in CPP disease, concluded that US can demonstrate CPP crystals in peripheral joints and that sensibility and specificity appear excellent and possibly better than those of conventional X-rays. This is the first time that US enters, even as a recommendation, in a diagnostic criteria set for CPP disease. Furthermore, the EULAR task force confirms that the absence of CPP in traditional radiology should not exclude the diagnosis and that definite diagnosis is by identification of CPP crystals in synovial fluid, but they also highlight that synovial fluid observers should be adequately trained as sensibility and specificity depend on the observers skills. 

Our opinion is that there is no universally acceptable gold standard for the diagnosis of CPPD [19], although there is some evidence that ultrasonography could be more sensitive than plain radiography for detecting CPP deposits [4, 20] and that synovial fluid analysis could be used as gold standard for daily routine diagnosis and probably for future studies. If this is true, US could be positioned at a higher step in the diagnostic procedure for CPP disease. In our previous study [11] we sought to assess the sensitivity and specificity of US in identifying CPP deposits in human menisci, comparing it to microscopy as the gold standard.

The sensitivity and specificity values obtained in that study are lower than those obtained previously by us [6] or other researchers [7]. We believe that this could be due to various reasons: firstly, the relatively small number of patients enrolled in this study; a larger number would probably have led to different results. Secondly, in this study we used microscopic analysis as the gold standard; some of these patients may have been negative at plain radiography or synovial fluid analysis and would therefore have been classified as normal if only conventional methods for diagnosis had been used (i.e., we found one patient with few crystals in only one meniscus). Furthermore, we examined only two menisci for each patient. Obviously, if we had examined the hyaline cartilage and both menisci of each knee, we would have had more likelihood of finding CPP deposits or excluding such a diagnosis, as we now know that CPPD is a polyarticular disease [21], thus increasing the sensitivity and specificity values. Finally, in this study we describe a new pattern of CPPD on menisci, an hyperechogenic line on the meniscus surface. Probably the research of this sign during in vivo analysis could have brought a further increase of sensitivity and specificity values. 

### 4.2. How to Improve the US Approach to CPP Deposits Identification in Fibrocartilage: Pitfalls and a New US Sign of CPPD

At the end of our study period we reviewed our data and tried to explain the main reasons for the false positive and false negative results. The typical aspect of CPP deposits in the joint fibrocartilage, described in previous works [4, 5], was found and confirmed in our study and should continue to be used. Below we discuss the main sources of error and pitfalls. 

**Figure 3:**
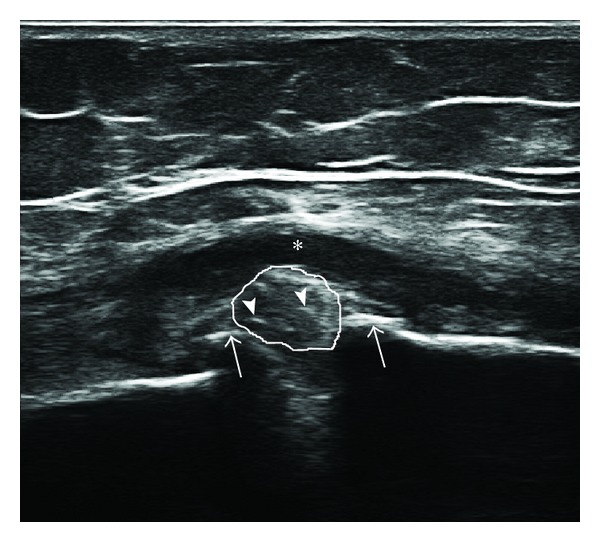
Medial meniscus of a subject with advanced osteoarthritis of the medial compartment of the knee. In this case we can observe parameniscal fluid collection (asterisk), osteophytes of both the femur and tibia, advanced meniscus degeneration with loss of the typical triangular shape of the meniscus (thin white line), and marked inhomogeneity of the US structure. In the meniscus we can observe thin linear hyperechogenic deposits (arrowheads) that could be due to CPPD deposits. In this case only a part of the medial meniscus could be adequately visualized, with the risk of missing other, more characteristic, deposits that could facilitate diagnosis. CPPD crystals were present in this meniscus, but we were not able to define whether these lines were due to CPPD crystals or not.

Firstly, our sample of patients was composed of subjects with late stage osteoarthritis. In this condition marked osteophytosis of the medial and lateral compartment of the knee is associated with a high degree of degeneration of the menisci, frequently associated with lesions and meniscal protrusion from the joint rim. Furthermore, elderly persons are often overweight meaning that it is not always possible to obtain sufficiently high-quality images to permit the correct evaluation of every detail. In such conditions the whole body of the meniscus is not always easy to identify and examine (Figure 3).

**Figure 4:**
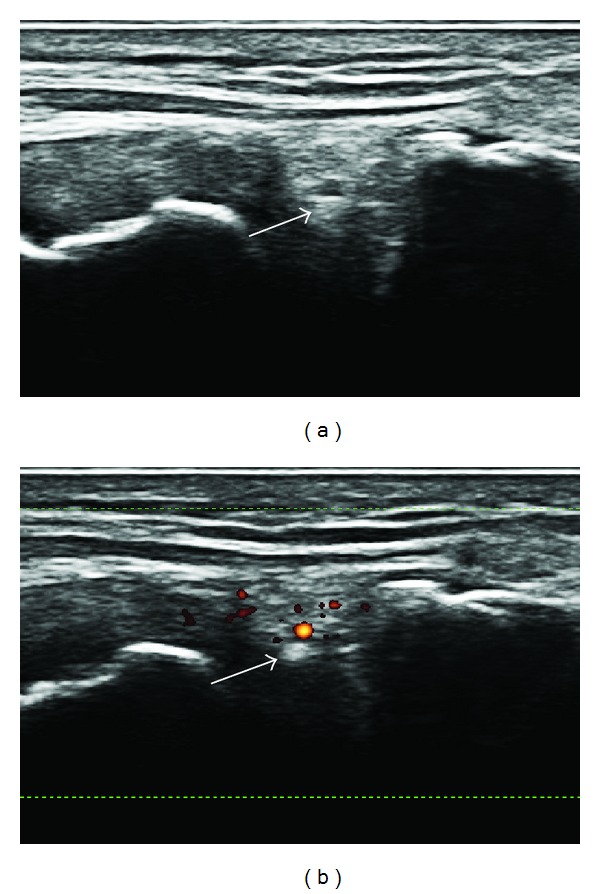
Small hyperechogenic deposit in a medial meniscus (arrow). Power Doppler US highlights the presence of a small vessel just above the focal hyperechogenicity, stressing the probability of posterior enhancement due to the presence of blood (water) in the vessel.

In some cases we found a small vessel in the meniscus. These vessels can create posterior enhancement due to the presence of water (blood) in their lumen and could create false positive images of CPP deposits. Power Doppler US could resolve this problem by highlighting the blood flow in the vessel (Figure 4). 

**Figure 5:**
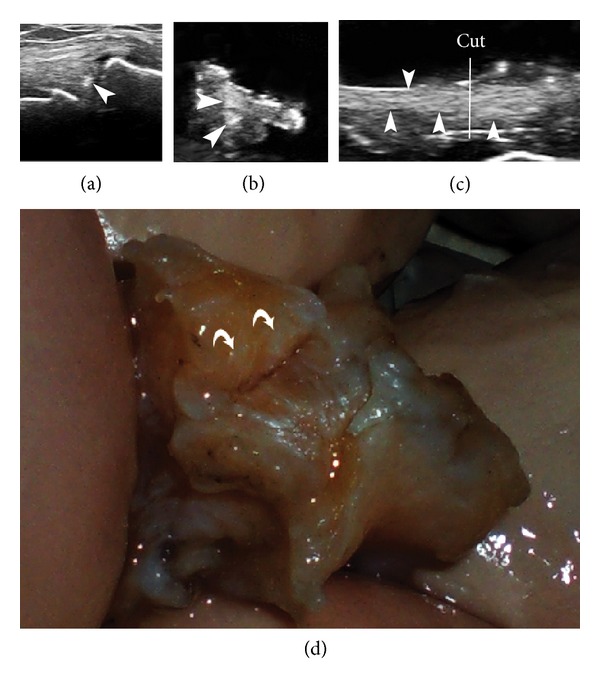
In vivo analysis (a), ex vivo analysis (b, c), and macroscopic analysis (d) of a medial meniscus. Note the presence of small round hyperechogenic deposits in a and b (arrowheads). The longitudinal scan of the meniscus in the ex vivo analysis highlighted the presence of hyperechogenic parallel lines (arrowheads) in the upper half of the meniscus, suggesting fibrous tissue, which in a transverse scan could resemble CPP deposits. The US guided cut (white line in panel (c) of the meniscus confirmed the presence of white lines and the absence of CPP deposits (macro- and microscopic).

Another confounding factor could be the difficulty of examining menisci using perpendicular scans. In fact, longitudinal scans are not usually used and are frequently not easy to obtain. In one case we were induced to give a positive result in a medial meniscus in the in vivo analysis, but the ex vivo analysis revealed that the hyperechoic deposits seen were due to the presence of fibrous tissue in the meniscus; this was confirmed by macroscopic examination and then by microscopic analysis, which did not demonstrate the presence of CPP crystals. The longitudinal scan of the meniscus clarified this case as it allowed us to identify long hyperechoic parallel lines in the mid-portion of the meniscus that in the transverse scan had the appearance of small round hyperechoic deposits similar to CPPD (Figure 5). In the in vivo examination, a continuous scan of the whole surface of the meniscus without raising the probe could make it possible to differentiate between a continuous hyperechoic line corresponding to fibrous degeneration and irregular, discontinuous CPP deposits.

 Furthermore, we are used to considering the hyperechoic deposits (not creating posterior shadow) that we find within the cartilage and fibrocartilage structures of the joints in US examination as CPP deposits [4, 5, 8]. In this study we found out that CPP deposits were common also on the surface of the menisci and not only inside them. These superficial deposits are more difficult to identify because of the presence of the interface between the meniscus and surrounding structures, such as hyaline cartilage or synovial fluid. Their identification is also problematic in ex vivo analysis, as they could appear as very thin hyperechoic lines, depending on the amount of crystal deposition. Dynamic scanning of the knee is very important in these cases, making it possible to understand whether these hyperechoic lines are in the hyaline cartilage surface (they move with the femur) or in the meniscus (they remain attached to the meniscus). The presence of these lines on the surface of the meniscus at US could be a new diagnostic criterion for CPP disease.

### 4.3. Further Analysis of the US and Microscopic Findings: New Pathogenetic Implications?

It is worth mentioning that even in the ex vivo analysis US could not be able to identify CPPD in 3 menisci. In these cases microscopic analysis demonstrated the presence of few CPP crystals in the specimens. To our point of view, this means that CPP crystal formation in fibrocartilage is a very slow process and imaging, and probably even microscopic analysis of synovial fluid, cannot detect the early phases of the disease when CPP crystal aggregates are too small to be visualized even with high-frequency probes. On the other hand, we should not fall in the trap to diagnose as CPP deposits every hyperechogenic spot of the meniscus as we risk to overdiagnose the disease. It is clear however that the disease's timeline and natural history is unknown and we are still armless before the challenge of an early diagnosis. We recently proposed a US scoring system that could help in the followup of patients affected by CPPD and contribute to the understanding of how crystals deposits change over the time, which factors could affect crystal deposition and possibly the role of a pharmacological treatment [21]. 

Furthermore, the fact that CPP crystals have been found on the surface of the menisci more frequently than in the fibrocartilage itself (9 versus 8) could open new pathogenetic scenarios. Traditionally, CPP crystals are believed to be produced within the cartilage due to modifications of the cartilage matrix and of the concentrations of calcium, magnesium, inorganic pyrophosphate, and other elements [9]. Some possible explanations for this fact are as follows: CPP crystals deposit on the meniscus surface after shedding from other anatomical structures or they emerge on the surface after being produced in the fibrocartilage, or they get produced directly on the meniscus surface. In this last case, intra-articular ambient and particularly synovial fluid composition could play a central role in CPP formation. Further studies on fibrocartilage and mainly on the hyaline cartilage could be useful to clarify this aspect. 

## 5. Conclusions

In conclusion, US can be considered a reliable technique for the identification of CPP deposits in menisci, as demonstrated by the ex vivo US examination, especially from the point of view of its high specificity. Much greater attention should be paid during in vivo examination, due to the presence of confounding factors, including the patients' characteristics and the presentation either of CPP deposits or the fibrocartilage itself, which require further study and assessment in order to avoid false positive and negative results. 

Finally, we would like to stress the fact that this study highlights the need of further studies in order to assess the real sensitivity and specificity of US in identifying CPP crystals versus a severe and definite gold standard in order to describe the reasons of false positive and negative results and to conform findings among different sonographers. We believe that the definition of an algorithm, intended as number of sites to be examined and US aspect of CPP deposits, could be very useful in order to optimize the sensitivity and specificity values of the technique. 

The design of this study is not targeted to the assessment of sensitivity and specificity of US in CPP disease, but it was meant to find and describe possible sources of error in identification of CPP crystals in fibrocartilage tissue. As mentioned previously, the possibility of assessment of various joints and anatomical structures during US examination permits to obtain high sensitivity and specificity values as described in previous studies [4, 6].

## References

[1] Coari G, Iagnocco A, Zoppini A (1995). Chondrocalcinosis: sonographic study of the knee. *Clinical Rheumatology*.

[2] Foldes K (2002). Knee chondrocalcinosis: an ultrasonographic study of the hyalin cartilage. *Clinical Imaging*.

[3] Falsetti P, Frediani B, Acciai C (2004). Ultrasonographic study of Achilles tendon and plantar fascia in chondrocalcinosis. *Journal of Rheumatology*.

[4] Frediani B, Filippou G, Falsetti P (2005). Diagnosis of calcium pyrophosphate dihydrate crystal deposition disease: ultrasonographic criteria proposed. *Annals of the Rheumatic Diseases*.

[5] Grassi W, Meenagh G, Pascual E, Filippucci E (2006). ‘Crystal clear’-sonographic assessment of gout and calcium pyrophosphate deposition disease. *Seminars in Arthritis and Rheumatism*.

[6] Filippou G, Frediani B, Gallo A (2007). A “new” technique for the diagnosis of chondrocalcinosis of the knee: sensitivity and specificity of high frequency ultrasonography. *Annals of the Rheumatic Diseases*.

[7] Filippucci E, Riveros MG, Georgescu D, Salaffi F, Grassi W (2009). Hyaline cartilage involvement in patients with gout and calcium pyrophosphate deposition disease. An ultrasound study. *Osteoarthritis and Cartilage*.

[8] Filippucci E, Scirè CA, Delle Sedie A (2010). Ultrasound imaging for the rheumatologist. XXV. Sonographic assessment of the knee in patients with gout and calcium pyrophosphate deposition disease. *Clinical and Experimental Rheumatology*.

[9] Ryan LM, McCarty DJ, McCarty DJ, Koopman WJ (1997). Calcium pyrophosphate crystal deposition disease, pseudogout and articular chondrocalcinosis. *Arthritis and Allied Conditions*.

[10] Zhang W, Doherty M, Bardin T (2011). European league against rheumatism recommendations for calcium pyrophosphate deposition. Part I: terminology and diagnosis. *Annals of the Rheumatic Diseases*.

[11] Filippou G, Bozios P, Gambera D (2012). Ultrasound detection of calcium pyrophosphate dihydrate crystal deposits in menisci: a pilot in vivo and ex vivo study. *Annals of the Rheumatic Diseases*.

[12] Ralph Schumacher H, Reginato AJ (1991). *Atlas of Synovial Fluid Analysis and Crystal Identification*.

[13] Punzi L (2009). *Manuale di analisi del liquido sinoviale*.

[14] Adinolfi A, Picerno V, Di Sabatino V (2013). Inquiry is fatal to certainty: is the US double contour sign specific for uric acid arthritis?. *Arthritis & Rheumatism*.

[15] Martínez Sanchis A, Pascual E (2005). Intracellular and extracellular CPPD crystals are a regular feature in synovial fluid from uninflamed joints of patients with CPPD related arthropathy. *Annals of the Rheumatic Diseases*.

[16] Schlesinger N, Hassett AL, Neustadter L, Schumacher HR (2009). Does acute synovitis (pseudogout) occur in patients with chronic pyrophosphate arthropathy (pseudo-osteoarthritis)?. *Clinical and Experimental Rheumatology*.

[17] Rosenthal AK (2007). Update in calcium deposition diseases. *Current Opinion in Rheumatology*.

[18] Abreu M, Johnson K, Chung CB (2004). Calcification in calcium pyrophosphate dihydrate (CPPD) crystalline deposits in the knee: anatomic, radiographic, MR imaging, and histologic study in cadavers. *Skeletal Radiology*.

[19] Filippou G, Frediani B (2012). The diagnosis of calcium pyrophosphate dihydrate crystal deposition disease: the good, the bad and… ultrasonography!. *Reumatismo*.

[20] Thiele R, Schlesinger N (2007). Ultrasound detects calcium pyrophosphate dehydrate crystal deposition in hyaline cartilage more readily than conventional radiography and MRI in pyrophosphate arthropathy. *Arthritis & Rheumatism*.

[21] Filippou G, Filippucci E, Tardella M (2013). Extent and distribution of CPP deposits in patients affected by calcium pyrophosphate dihydrate deposition disease: an ultrasonographic study. *Annals of the Rheumatic Diseases*.

